# Vehicular Platoon Communication: Architecture, Security Threats and Open Challenges

**DOI:** 10.3390/s23010134

**Published:** 2022-12-23

**Authors:** Sean Joe Taylor, Farhan Ahmad, Hoang Nga Nguyen, Siraj Ahmed Shaikh

**Affiliations:** 1 Centre for Future Transport and Cities (CFTC), Coventry University, Coventry CV1 5FB, UK; 2 Volta Trucks, Wokingham RG41 5TP, UK; 3 Systems Security Group (SSG), Department of Computer Science, Swansea University, Bay Campus, Fabian Way, Swansea SA1 8EN, UK

**Keywords:** platoons, intelligent transportation systems, security, cyber-attacks, smart cities, communication security

## Abstract

The emerging technology that is vehicular platooning is an exciting technology. It promises to save space on congested roadways, improve safety and utilise less fuel for transporting goods, reducing greenhouse gas emissions. The technology has already been shown to be vulnerable to attack and exploitation by attackers. Attackers have several attack surfaces available for exploitation to achieve their goals (either personal or financial). The goal of this paper and its contribution to the area of research is to present the attacks and defence mechanisms for vehicular platoons and put risks of existing identified attacks forwards. Here the variety of attacks that have been identified in the literature are presented and how they compromise the wireless communications of vehicle platoons. As part of this, a risk assessment is presented to assess the risk factor of the attacks. Finally, this paper presents the range of defence and countermeasures to vehicle platooning attacks and how they protect the safe operations of vehicular platoons.

## 1. Introduction

Vehicular Platooning technology has developed to a point where there is starting to be limited and controlled deployment of this new technology [1]. Vehicular platooning technology promises to improve road safety, reduce fuel consumption, traffic congestion, and CO_2_ emissions by making use of wireless communications and semi-autonomous driving [2,3]. To operate, vehicular platooning relies on two main technologies; (1) Cooperative Adaptive Cruise Control (CACC) [3], and (2) deployment of Vehicular Ad hoc NETwork (VANET) [4]. CACC is a cooperative extension of adaptive cruise control, which enables the vehicles to form a platoon [3]. When vehicles are platooning, the Vehicle-to-Vehicle (V2V) communications of VANET are used to exchange various information such as vehicle location, speed, and acceleration, to name a few.

VANET is not just V2V communications. A vehicle within the VANET network will be communicating with a wide range of different nearby nodes such as pedestrians, cameras, and other adjacent infrastructure usually known as Roadside Unit (RSU) [5]. When a vehicle connects to an RSU, the communication is called Vehicle-to-Infrastructure (V2I) communications [6]. RSUs can link together vehicles that cannot directly interact with each other and act as middle-man between platoons and platoon service providers. This ability of the vehicles to communicate with a range of different systems via various communication modes is also referred to as Vehicle-to-Everything (V2X) communication [7].

A vehicular platoon is an application of VANET. All members work cooperatively to travel in an extremely close formation to a common destination. The basic makeup of a vehicular platoon is a platoon leader, under the complete control of a trained and experienced driver, and member vehicles. Member vehicles during vehicular platooning are driven autonomously or semi-autonomously using sensor information and instructions from other vehicles in the platoon, particularly the leader [8]. Wireless communications are used to link vehicles together, so they act as one. To achieve this, the wireless links exchange information from the leader and other members, such as speed, position, and acceleration, to name a few parameters [2]. The details are explained in detail in Section 2.

### 1.1. Motivation for Vehicular Platoon Cybersecurity

As was said initially, this technology is just starting to see some deployment on our roadways [1]. While it will be some time yet before platoons are common sights on our roadways, it will become so very soon. Governments seek to improve traffic flow and reduce the carbon footprint of their countries. In addition, companies are always looking at ways to reduce operating costs and improve their public image, with low environmental impact becoming more and more attractive to consumers. The combination of reducing environmental impact and operating costs is a way for companies to continue to be competitive in the years to come.

As this technology becomes deployed, it will face many challenges, one of which will be cyberattacks. Adversaries will seek to cause disruption for users for a multitude of reasons, such as peer recognition, financial gain, and personal grudges. It is, therefore, vital to protecting vehicular platoons from cyberattacks as much as possible.

Platooning relies heavily on wireless communications between members to provide digital links between members. These digital links are used to maintain the structure and safety of the platoon. Due to the nature of wireless communication and the beacon broadcasting used by platoons, digital links are vulnerable to various cyber-attacks. More specifically, the radio wireless signals of the IEEE 802.11p standard have shown to be vulnerable to a range of attacks such as jamming attacks [9], Sybil attacks [10], and ghost vehicles attacks [11], to name a few.

When vehicular platoons are attacked or compromised via wireless communications, the vehicular platoon can no longer function correctly. This can lead to a range of problems, such as failing to maintain a steady inter-vehicle distance, the theft of information about the vehicular platoon or platoon member, and preventing members from joining or being part of a vehicular platoon. For a company, individual or government, this is a problem. First, there are massive safety issues with this as the vehicles can collide with each other, other traffic, or infrastructure due to missing, delayed, or fake information. The second is that the benefits of vehicular platooning are lost during an attack. A vehicular platoon or platooning-enabled vehicle cannot maintain the tight formation required for safe operation. For example, a platoon cannot continue to operate in the presence of a jamming attack. Third and finally is that information about the vehicular platoon members can be stolen.

### 1.2. Aims and Contributions

The major contributions of this literature review are threefold: (1) To discuss the cyber security aspects of vehicular platoons by presenting the current threat landscape on wireless communications within vehicular platoons. (2) To provide an insight into the risk of attacks on the vehicular platoon by introducing risk assessment according to SAE/ISO 21434 standard [12], (3) To provide solutions to the identified attacks for vehicular platoons and at the end present the open challenges facing the cybersecurity aspects of wireless communications in platoons.

### 1.3. Existing Surveys and Contribution of This Paper

Table 1 provides a comparison of survey papers from 2015–2022 discussing vehicular platoons. It shows that these survey papers have partially discussed the cyber security issues of vehicular platoons. To this end, this paper aims to include the broadest and most variety of attacks proposed in the literature and the countermeasures that can be deployed to counter the attacks. In addition, we will add a risk assessment for the attacks on platoons. The goal of the risk assessment aimed to help understand what types of attacks are likely to be the most damaging to the operation of vehicular platoons.

The surveys in Table 1 discuss attacks to platoons and VANETs Out of those papers that discuss attacks on platoons directly, only [16,19] discuss the attacks specifically for vehicular platoon application. The other papers tend to be more CAV and VANET. While platoons are a type of CAV, they are different as platoons rely far much more on the wireless communications link to maintain safe driving conditions. In addition to providing a comprehensive look at platoon attacks and platoon defences, this review also presents the risks presented to vehicle platoons from the identified attacks using the SAE/ISO 21434 standard [12].

Paper [19] provides a detailed explanation of various attacks as well as the defence mechanism on vehicular platoons, which are primarily identified from the previous literature. When discussing the attacks and how they compromise platoons, each attack is linked to both the network layer that targets the attack and the security attributes that the attack compromises. The attacks, however, are restricted to just V2V communication between platoon members with no consideration to attacks to the broader V2X environment.

When surveying platoons and the range of attacks that can compromise them, few others directly talk about the attacks. The authors in [14] surveys platoon security; however, there is only very limited discussion on the attacks that pose a threat to platoons as the paper’s primary focus is the cyber-physical systems for platoons. While this provides a valuable discussion of how attackers can get access to a platooning vehicle, there are no extensive discussions for attacks.

Platoons are a type of Connected and Autonomous Vehicles (CAV), even if platoons are far more reliant on information flow than standard connected vehicles. In a platoon, if the communications are blocked or inaccurate due to any unforeseen circumstances, it can lead to a major incident. It is, however, reasonable to consider that many of the identified attacks seen for CAVs can also be applied to platoons: Sybil, Replay, and Black Hole, to name a few. Currently, there are a variety of papers that discuss various attacks and defence mechanisms specifically for CAV [13,14,15,17]. However, these papers provide very little or no direct discussion about platoons cyber security. However, the attacks are still relevant as platooning is an application of CAVs.

### 1.4. Paper Organization

The rest of the paper is organised as follows: Section 2 will introduce vehicle platooning and explain the architecture in detail. In addition to this, the Wireless Access for Vehicular Environments (WAVE) architecture will be described to help the discussion on the risks to vehicle platoons. In Section 3, a variety of attacks are presented, and we explore how attacks can be grouped together. Section 4 will present the threat landscape where attacks on the platoons are discussed in detail and categorised, which are categorized based on the cybersecurity attributes. After identifying the attack and how it can compromise a platoon, the following Section 5 will use the SAE/ISO 21434 standard [12] to create a risk assessment. In this Section, the attacks identified in Section 4 will be assessed for the risk that they pose to vehicular platoons. After identifying the risks of the attack, Section 6 will discuss the range of proposed defences to prevent the attacks discussed above. Moreover, Section 7 will provide future challenges and gaps in existing knowledge, and, finally, we conclude the paper in Section 8.

## 2. Introduction to Vehicular Platooning, Communication Topology and WAVE Architecture

**Figure 1:**
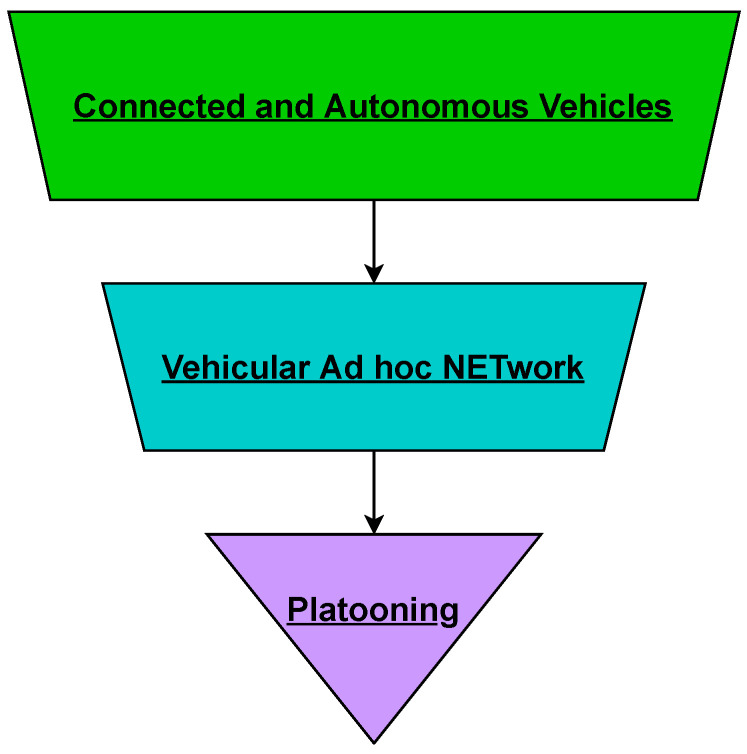
A visual representation of relationship between CAV, VANET, and Vehicular Platoon.

As described earlier, vehicular platoons are a group of linked vehicles using digital links, which enable the lead vehicle to dictate the movements and behaviour of all members. Platooning can be described as an application of VANET where a manually driven vehicle enables other vehicles’ to autonomous or semi-autonomous drive [21]. In a platoon, the lead vehicle driver becomes responsible for driving the entire platoon and can behave like a single vehicle. Such manoeuvres can be achieved through using wireless communications to transmit control information to other platoon members [22]. Figure 1 shows the relationship between CAV, VANET, and platoons, with VANET being one application of CAV and platooning being an application of VANET.

**Figure 2:**
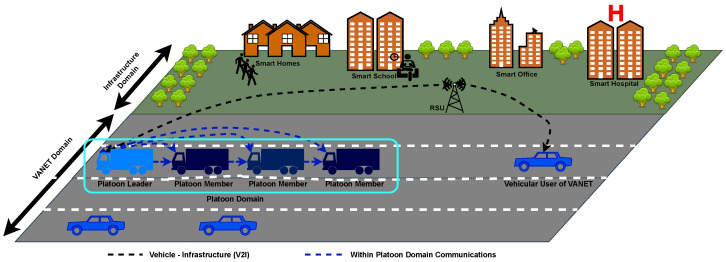
Current Example of Vehicular Platooning within a Smart City.

Figure 2 provides an illustration of vehicular platooning within the broader context of smart cities-based CAV and VANET environments. Here it is shown that the platoon operates within the platoon domain—the platoon as an entity communicates to the broader VANET environment. In the VANET environment, other CAVs can be connected to infrastructure such as RSUs and other potential connected road users such as pedestrians [23].

### 2.1. Vehicular Platoons

A vehicular platoon is made up of three types of vehicles.

1. *Lead Vehicle:* Mostly driven manually to observe the environment more accurately.
2. *Member Vehicles:* driven autonomously or semi-autonomously, with their drivers simply monitoring their vehicle’s systems [24], and finally,
3. *Join/Leave Vehicles:* which transition into or out of the platoon. When they are transitioning into the platoon, they are driven normally until it is safe for them to switch to autonomous driving [8]. On the other hand, when the vehicle is transitioning out of the platoon, it is autonomously driven until the system tells the driver it is safe to take over [8].

Platoons use beaconing to maintain formation. Beaconing is where each vehicle transmits information related to the *position, speed, acceleration, target speed and or acceleration, vehicle ID, membership status, and travel direction* to all other member vehicles [13,25,26]. This behaviour enables the platoon to have improved safety due to a dramatically reduced reaction time to any action undertaken by the leave vehicle and reduces the workload on drivers of the member vehicles [27]. The member vehicle can handle most driving situations using the instructions given by the lead vehicle [19]. In some platooning setups, this can mean that member vehicle drivers are only monitoring their vehicle systems [8]. When forming platoons, vehicles may take a slight detour to be able to meet another platooning-enabled vehicle [28]. The next subsection will break down the advantages of platoons in depth.

### 2.2. Advantages of Platooning

There are two main advantages to platooning technology; these are reduced inter-vehicle spacing and ensure traffic safety. In addition, by reducing the inter-vehicle spacing, the fuel economy of the vehicles involved will also improve, in some cases dramatically. By improving the vehicle’s fuel economy, the running costs are also significantly reduced, as well as the vehicle’s output of harmful greenhouse gasses [29,30,31].

#### 2.2.1. Inter-Vehicle Spacing

**Figure 3:**
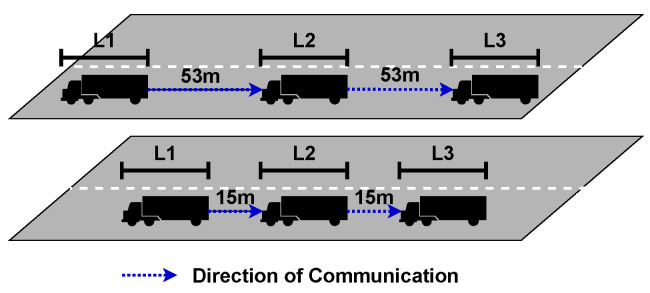
Platoon inter-vehicle space compared to non-platooning vehicles.

The wireless communications enable the platoon members to drive significantly closer to each other safely compared to regular driving. In platoon applications, an inter-vehicle distance of 15 m is used due to safety but a theoretical possible gap as small as 7 m at speeds up to 80 km/h [2]. In comparison, in the United Kingdom, the safe inter-vehicle spacing is recommended to be the braking distance which for a standard vehicle is 53 m [32]. This distance is, however, for Heavy Goods Vehicles (HGV) such a Lorry’s can be significantly more, up to 40% [33]. Therefore, reducing the safe inter-vehicle distance for each vehicle will significantly reduce road space used by platooning vehicles. An example of a simple, three-vehicle platoon compared to three non-platooning following vehicles considering the inter-vehicle of 53 m is shown in Figure 3. Here, three non-platooning vehicles with a total minimum footprint of $L_{1}+53+L_{2}+53+L_{3}$ where $L_{i}$ is the length of the vehicle. A three-vehicle platoon with an inter-vehicle distance of 15 m will be instead $L_{1}+15+L_{2}+15+L_{3}$ again $L_{i}$ is the length of each vehicle. The inter-vehicle distance between two consecutive vehicles is 53 m. In this scenario, the total inter-vehicle distance among the three vehicles is 106 m, with each vehicle maintaining a distance of 53 m with the vehicle in front. In contrast, a three-vehicle platoon cuts this down to just 30 m, with an inter-vehicle distance of 15 m.

#### 2.2.2. Fuel Economy

The reduction to inter-vehicle space means that the drag forces from air resistance are significantly reduced [34]. Vehicle engines will work less and therefore consumes less fuel for a journey compared to the same journey but without using platooning. The reduction in fuel consumption can be up to 9.7% in the member vehicle, and up to 5.3% for the leader in a platoon of two vehicles, thus resulting in the overall fuel savings being between 3.7% and 6.4% [35]. These values can vary due to the type of driving the vehicle does. When a vehicle is idling and or accelerating often, then the efficiency overall will reduce. Platooning vehicles can maintain the formation without regular braking and acceleration; this will naturally also improve fuel economy. By reducing the amount of fuel used, the vehicle will be able to travel much further without needing to stop for more fuel. Refuelling less often, truck operators will save money that would have otherwise been spent on fuel [22].

#### 2.2.3. Environmental Impact

Apart from saving fuel, by reducing fuel consumption, the vehicle will also see a reduction in CO_2_ output and other greenhouse gasses [36]. This is important as there is a collective push to reduce greenhouse gas emissions by businesses, consumers, and governments. Currently, in the UK, it is assumed that, on average, with average size load, an HGV will output 0.85049 kg/km of CO_2_ [37]. This value is highly volatile as the amount of CO_2_ produced will change depending on the type of driving the vehicle, its average speed, and fuel consumption. Having a drop of around 5% over a single journey will have a small but meaningful impact on the surrounding environment [35]. The slight reduction can quickly snowball into a far more considerable reduction over an entire fleet of vehicles [22].

#### 2.2.4. Traffic Safety

Worldwide, there is a drive to improve safety on roadways, with it estimated that 90% of road accidents are attributed to human error [22]. With there around 1.25 million fatalities on roads worldwide each year [38] and a further 20 to 50 million people suffer from non-fatal injuries, with many leading to disability each year [38]. The World Health Organisation (WHO) also believes that by 2030 road accidents will be the seventh leading cause of death without sustained action [38]. Not only does this have an impact on human loss, but road accidents also have a financial impact. The WHO estimates that for many counties, this could be as much as three percent of their gross domestic product could be lost through road traffic incidents [38]. Platooning helps to remove human error from driving as all braking and acceleration is controlled by the lead vehicle driver, and there is, therefore, almost no delay between braking and reaction between platooning members.

### 2.3. Communication Topology

**Figure 4:**
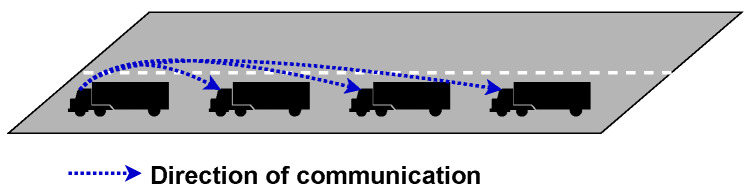
Centralised topology of platooning communications.

**Figure 5:**
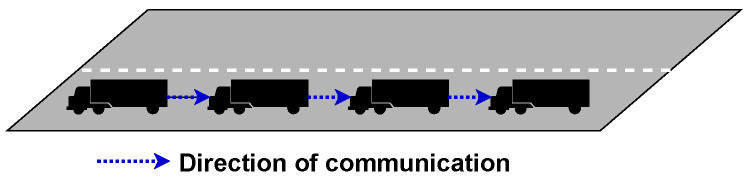
Decentralised topology of platooning communications.

**Figure 6:**
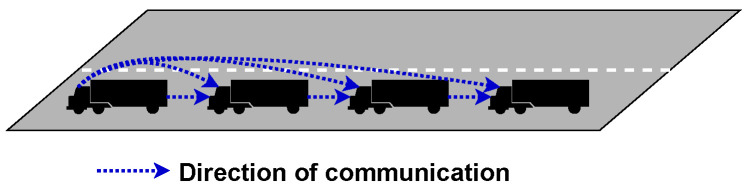
Predecessor-leader following topology of platooning communications.

**Figure 7:**
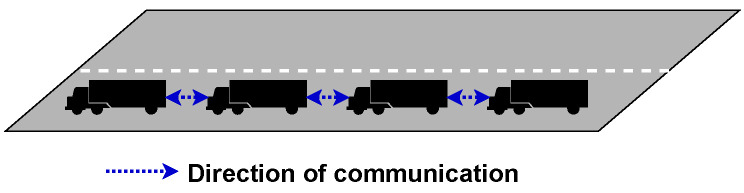
Predecessor-leader following topology of platooning communications.

**Figure 8:**
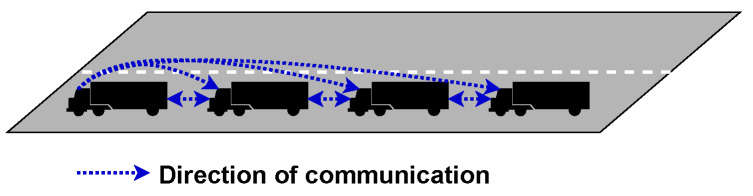
Bidirectional-leader topology of platooning communications.

**Figure 9:**
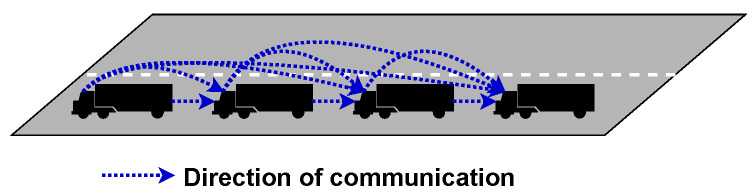
Two-predecessors following the topology of platooning communications.

Many wireless communication typologies can be seen implemented in platooning [8,19,39]. Each topology comes with different advantages and disadvantages. Overall, the goal is to create a stable network that creates a stable platoon where information can quickly and reliably be transmitted to all members. This has resulted in the creation of three topologies: Centralized, Decentralized, and Hybrid, which are much visually shown in Figure 4, Figure 5, Figure 6, Figure 7, Figure 8 and Figure 9, respectively.

#### 2.3.1. Centralized Topology

Centralised topology is where the leader communicates with all vehicles in the platoon; however, member vehicles do not communicate with any vehicle in the platoon. This leaves the leader in sole control of the platoon. This approach is advantageous at quickly making all members aware of actions done by the leader but leaves them without information about other platoon members. There is still communication with the leader, but it is only GPS and the vehicle’s velocity, which is done periodically. The leader will then decide what to do with the information and then transmit individual commands to each vehicle. The challenge with this method is the high number of packets that are being transmitted within the platoon, which can produce a significant transmission delay. As a result, packets are usually received by the members very late and outside of tolerance limits. This topology is shown in Figure 4, highlighting that only a leader can communicate with its platoon members.

#### 2.3.2. Decentralized Topology

In decentralised topology, each vehicle communicates with the vehicle directly behind them. With this topology, the lead vehicle is doing significantly fewer computation tasks. In addition, packets are less likely to be significantly delayed as fewer packets are transmitted to maintain platoon stability. The challenge with this topology is that it can create instability within the platoon when vehicles are leaving and joining. When a vehicle leaves a platoon and is not the rear vehicle, it can create a connectivity hole that will need to be closed. When a vehicle joins or leaves, member vehicles will need to sense and adjust their velocity to maintain platoon stability quickly. This topology is presented in Figure 5.

#### 2.3.3. Hybrid Topology

For hybrid topology, there are four main ways that centralised and decentralised topologies can be combined. Each method has its own merits and disadvantages and seeks to overcome the problems of just using a single topology. These topologies are (a) Predecessor-leader following, (b) Bidirectional, (c) Bidirectional leader, and (d) Two-predecessors following.

*Predecessor-leader following* works by having the leader transmitting to all vehicles, and each vehicle communicates with the vehicle directly behind it, as shown in Figure 6. This topology, along with *Bidirectional* topology, was designed to take advantage of Cooperative Automated Cruise Control (CACC). Using CACC, far more information can be passed between members and used by the vehicle’s onboard computer to make better decisions.

*Bidirectional* topology is when each vehicle can send and receive messages from neighbouring vehicles as depicted in Figure 7. The advantage to this is that information from members can flow both ways, which is helpful as environmental sensor and vehicle information can be passed to all members. An example of when this would be useful is when a car overtakes the platoon. The vehicle at the rear can inform all member vehicles that the vehicle is approaching.

*Bidirectional-leader* takes Bidirectional along with Centralised to create a topology that seeks to overcome the weaknesses of both methods. By having the leader control the platoon size and stability, the members can communicate directly between themselves, as is shown in Figure 8.

*Two-predecessors following* is an advancement on predecessor-leader following to give vehicles better awareness of what other vehicles are doing without increasing the number of transmitted packets and is shown in Figure 9. This can help improve situational awareness; however, it will require far greater processing power to process and act on all this additional information quickly.

### 2.4. WAVE Architecture

**Figure 10:**
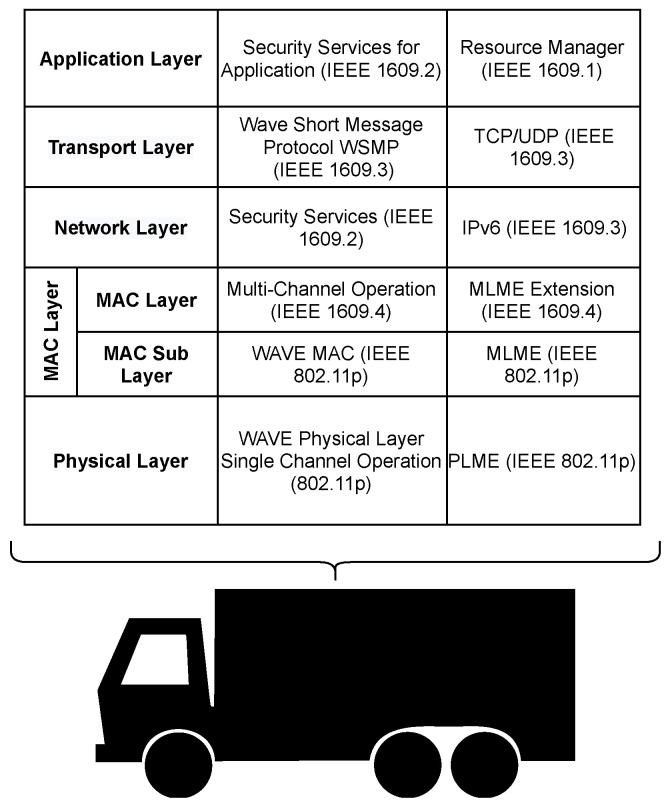
WAVE network stack.

Wireless Access in Vehicular Environment (WAVE) is used for all wireless communications for connected vehicles [40,41]. WAVE is built upon Dedicated Short-Range Communications (DSRC) based on the IEEE 802.x family [42] of standards. DSRC uses Carrier Sense Multiple Access with Collision Avoidance (CSMA/CA) that operates between 5.850 GHz to 5.925 GHz and, as defined in IEEE 1609.4 [41,43]. The stack is also shown in Figure 10. DSRC spectrum is formed using seven channels, one central Control Channel (CCH), and six Service Channels (SCHs). In addition, DSRC supports channel switching and simultaneous access of CCH, and SCHs [40,41], which is achieved as each channel is a 10 MHz band allocated to each channel. Each channel is separated by a 5 MHz guard band [40,41].

DSRC describes how the communication stack should be arranged, for example thus how tasks like addition and removal of frame headers and security measures. WAVE uses the IEEE standard, i.e., IEEE 802.11p, which was created explicitly for vehicle networks [41]. IEEE 802.11p is only used in the communication stack’s physical and data link layers; the rest of the stack is handled by IEEE P1609 standard [44]. WAVE can form networks both with and without IP as it can use WAVE Short Message Protocol (WSMP) in non-IP applications, which is inherited from DSRC.

To enable the multi-channel operation of WAVE, IEEE standard 1609.4 is used to manage the time between the SCHs and the CCH. IEEE standard 1609.3 specifies WSMP with the definitions for the inclusion of User Datagram Protocol (UDP), Transmission Control Protocol (TCP), and IPv6 within the system; these are taken from DSRC. The defining of these management functions is necessary for being able to provide network services. Further, the IEEE standards 1609.2 and 1609.1 are used. The 1609.2 standards describe the security service block for the protocol, and 1609.1 describe the resource manager.

WAVE is applied to vehicular platoon members. It defines the communication message steps between all vehicles in a platoon regardless of their role in the platoon. The WAVE network stack seen in Figure 10 is used to handle all communications between members [45]. The WAVE stack is also used in other V2V applications such as VANET and, as such, also enables platooning, giving enabled vehicles the ability to communicate with other CAVs [40,41].

**Figure 11:**
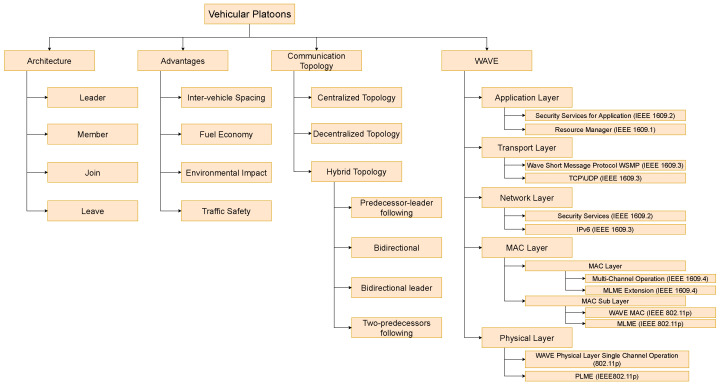
A detailed taxonomy of Vehicular Platoons.

Figure 11 is a visual breakdown of this section showing the different vehicle designations within vehicular platoons, the advantages of platooning, the different communication typology’s used in Vehicular platooning, and then WAVE itself and its components.

## 3. Vehicular Platooning Security Threats

Vehicular platooning security threats are wide-ranging and diverse. In this paper, only threats involving wireless communications will be investigated and such physical threats to the vehicle will not be considered. Even within this now smaller list of threats, there is a great range of attacks—all with their own goal results. These goals can be broken down into five distinct categories: (1) To prevent the formation of platoons, examples of this are Jamming, Denial of service (DoS), and Malware attacks. (2) Access management of platoons, where the attacker will represent another individual to manipulate vehicles within a platoon. Examples of such attacks are Impersonation, Reputation, and Illusion attacks. (3) Financial gain, where the attacker will seek to profit from the attack directly. This can be done by Eavesdropping on financial transactions or by tricking or forcing victims to pay using malware or ransomware. (4) Data Collection is when the attacker takes information from the wireless communications. Such attacks target the flow of information to, from, and between? platoon members. This information can then be sold or used for other purposes; examples are Eavesdropping and location disclosure. (5) Finally, there is the largest category which is platoon disruption. These are attacks that are predominantly used to disrupt a platoon and cause it to lose effectiveness or even unsafe. Examples of such attacks are; tunnelling, collision, and many other attacks.

There is a range of different cybersecurity threats that vehicular platoon communications are facing [46]. Some of these threats are direct, whose sole aim is to disrupt, or damage a platoon’s integrity to make it less efficient and cause discomfort to passengers. In contrast, few attackers will seek to break up or prevent platoons’ formation. Other attacks could be more subtle and seek to steal information about the users, vehicle, and load. There is a range of different ways that attacks on vehicular platoons can be classified. One common approach is cryptography-related classification [17,47]. Another approach for sorting attacks is by the layer they target in the communication stack, like in [19].

In this paper, while the security requirements for platoons are presented, the attacks are grouped into each attack’s goal. This can then be used to help to justify the risk of such an attack as discussed in Section 5. Table 2 presents each attack identified in the literature, the goal of the attack and the security requirement that is broken.

### 3.1. Authentication

Authentication is one way that is used by security mechanisms to prove or give credibility to a message. Authentication can be done in a wide variety of ways, such as with security certificates or the use of distinctive markers. These markers validate who the sender is and that they have permission to communicate. When an attacker breaks this, it often involves the use of stolen or fake security identifiers.

### 3.2. Availability

Availability in platooning network is the ability for members to connect, form, and maintain a network as well as their ability to maintain access to information and data. Availability needs to be maintained at all times ideally. However, there are times when availability may degrade naturally, such as adverse weather and physical barriers such as tunnels. An attacker can compromise the availability of a platoon network with jamming attacks or by the more well-known Denial-of-Service (DoS) attack [49].

### 3.3. Confidentiality

Confidentiality in a platoon network refers to only network members or authorised members who can decrypt messages that are broadcast from the leader or fellow platoon members. This can mean that if a platoon member wants to transmit to a specific vehicle, the information is only read by the target vehicle and not all other nodes of the Vehicular platoon network or wider CAV members.

### 3.4. Data Verification

The use of data verification is to constantly check data using multiple messages and/or the use of sensors. This is useful to check that the messages which propagate through the platoon domain are correct and, therefore, ensure the high integrity of the platoon.

### 3.5. Integrity

Integrity is where the reliability of the information is assured and that there has been no tampering with the message and the message content is accurate. When an attacker compromises the integrity, there can be no way to guarantee the reliability or accuracy of the received communication without additional information [59].

### 3.6. Privacy

Privacy in any network is essential, and platoons are no different. For platoon networks, users and their vehicles should only expose or give away unnecessary information to enable platooning. This information must also be treated with care by all parties involved. Any and all information should be destroyed after it is used and only kept for as long as it is needed. In addition, where possible, all information should be shared anonymously.

### 3.7. Non-Repudiation

Here when a message has been received, the sender cannot deny the message and must take responsibility for the message [60]. This can be done by using a secure black box recorder-type device that is used to solve incidents and disputes.

## 4. Platooning Communication Security Attacks

In this section, we provide details of various attacks that can affect platoon wireless communication networks. Each attack is sorted and categorized by the target outcome or goal of the attack. Next, a detailed discussion about the realization of attack within platoons is provided in this section. In Table 2, each attack is shown along with the goal of the attack and the security requirement that is broken CIA.

**Figure 12:**
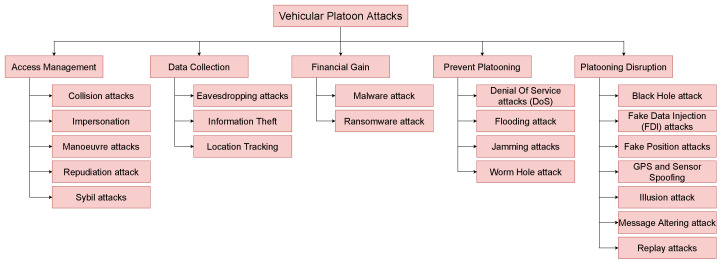
Attacks on platoons sorted in accordance with the intended outcome of the attack.

In this paper, attacks on platoons are grouped by the intended outcome of an attack as shown in Figure 12. The outcomes of attacks on platoons can be summarised as Access Management, Data Collection, Financial Gain, and Preventing Platooning. The reason for group attacks like this is to group attacks with similar impacts in a vehicular platoon. By sorting vehicular platoon attacks in this manner, researchers can quickly understand what attacks have similar goals and, therefore, impact a platoon. However, grouping attacks using the security attribute highlights network vulnerabilities.

### 4.1. Access Management

An Access Management attack is where attackers seek to manipulate access to the vehicular platoon or platooning service. This can be achieved in many ways and includes attacks such as Impersonation, Sybil, and Manoeuvre attacks, to name a few.

#### 4.1.1. Collision Attacks

In a collision attack, the attacker seeks to force packet collisions, which will result in the dropping of packets [19]. This will result in members not receiving packets resulting in an integrity violation of the information transmitted as discussed in the previous section [19]. Such an attack can be used to prevent some or all traffic by a platoon, and the attacker is selecting and choosing which node can communicate and when.

#### 4.1.2. Impersonation

An Impersonation attack is where a malicious node pretends to be another node. To do this an attacker needs to obtain the ID of another vehicle. As such an Impersonation attack compromises the integrity of messages in the platoon system. Whatever the malicious node does, others will think it is the user who had its ID copied [54]. Using a stolen ID can enable users or vehicles that are not paying for platooning service, banned, or poorly rated drivers to access the platooning service [54]. The impersonated user will see increased usage of their account from when the malicious node is impersonating them. There is also the potential for sudden dropouts from the platoon service provider being confused by two identical IDs being in use at once. The attacker is also able to commit other attacks without fear of reprisal while using a cloned ID [54]. All reprisals for the attackers’ actions are taken by the cloned vehicle [54].

#### 4.1.3. Manoeuvre Attacks

Platoon Manoeuvre attacks are fake entrance, fake leave, and fake split requests [51]. Fake entrance attacks can lead to gaps in platoons as members may open up to let new vehicles in or leave space for non-existent vehicles [39]. In addition, this can reduce the number of member vehicles that can join the platoon as the leader thinks more vehicles are part of the platoon than there actually are [39].

Fake leave and split requests can cause platoons to break up, which will decrease the efficiency of the platoons even more [39]. In this case, the attacker can take advantage of this to become the leader to target and deny specific vehicles access to the platoon [39]. This can then lead to a denial of service attack on vehicles. Overall Fake Manoeuvre attacks damage the Integrity and Availability of security characteristics.

#### 4.1.4. Repudiation Attack

With Repudiation attacks, the attacker attempts to confuse the network by denying that they have received messages when there is any dispute over messages [61]. In platoons, it is believed that this can cause the system can assign the same identity to multiple vehicles [19]. During this attack, this makes it almost impossible for network members to distinguish between members [19]. Furthermore, it enables the attacker to pretend to be other vehicles and manipulate the platoon [19].

#### 4.1.5. Sybil Attacks

Sybil attacks [62,63] are committed by malicious nodes that create one or more manufactured vehicles upon entering the platoon network and try to have these ghost vehicles accepted into the platoon [10,54]. When the ghost vehicles are part of the platoon, they can destabilise the platoon by creating gaps. The leader will also think there are more vehicles than there are, stopping new vehicles from joining. The attacker can take it a step further and try to take control of the platoon off the leader using the ghost vehicles [10,54]. Overall, Sybil attacks break authentication as nodes cannot differentiate ghost vehicles from real ones.

### 4.2. Data Collection

In Data Collection attacks, the attacker will target the message when transmitted between nodes to extract some useful information about the vehicular platoon or vehicles in the platoon. The information can then be used or passed on to others.

#### 4.2.1. Eavesdropping Attacks

An Eavesdropper listens and logs the communications of a network [50]. In platooning, this means that the attacker can see the beacon that members use to maintain the formation. If the network uses any form of encryption, the attackers will also need to decrypt the message to understand the communicated data. The primary goal of this attack is to gain information about the platoon and the member vehicles [50].

The attacker can use the information acquired to carry out another attack, such as Replay or Sybil, by knowing how the platoon needs beacon information and how to make the fake messages look authentic to the platoon. In addition, it may show a wide variety of different aspects about the platoons’ plans, such as rest stops and where vehicles plan to split up.

#### 4.2.2. Information Theft

As a rule of thumb, information is extremely valuable. However, for platoons, the information is very important as it contains sensitive information which can be gathered and used both legally and illegally [15]. When a vehicle is in a platoon network, it will be transmitting a multitude of information. The members will be transmitting information by the beacon to other members, including status updates and routes to vehicle-enabling platforms via RSU and GPS pings. This information can be used in various ways, both to improve the platoon service or to target individual vehicles by criminals [15]. Platooning enabling companies may sell some information to third parties to enable them to better target drivers with advertising; a current issue is who owns all this information: the driver, the fleet manager, the platooning enabling company, or another entity [15]? Not understanding who is legally responsible can lead to data leaks.

#### 4.2.3. Location Tracking

Location tracking attacks are where the attacker can track the position of a vehicle. This can be done in one of two ways. The first is by intercepting the GPS location information of a vehicle, and the second is by extracting it from the beacon. When intercepting the information from the GPS, an attacker is merely eavesdropping on the communications between the vehicle and the GPS satellites overhead. This type of attack is breaking the privacy of the attacked vehicle. When the location information is extracted from the beacon, this breaks down confidentiality between platoon members. All members need to remain anonymous in platoons, and only the intended target uses their location information.

### 4.3. Financial Gain

In a financial gain attack, the attacker will seek to directly steal or obtain financial information from the attacked platoon, vehicle, or service provider. During an attack for financial gain, the attacker will compromise the confidentiality of the network or vehicle.

#### 4.3.1. Malware Attack

Malware attacks on platoons can have catastrophic consequences to platoons, as they can shut down the whole network. Malware attacks have the potential to prevent users from platooning and even potentially using affected vehicles. Malware can be used for various attacks such as data collection, platoon destabilisation, and even ransoming vehicles or the network. In such attacks, while any security requirement can be broken by a malware attack, in most cases, Availability, Confidentiality, and Privacy are broken.

The malware first needs to infect a vehicle’s On-Board Computer; this can be done by connecting an infected device to a vehicle. CAV have many interfaces that an attacker can use to get the malware onto the vehicle [15]. These interfaces are; the On-Board Diagnostic (OBD) port, CD drive, USB interface, Bluetooth, and the wireless communication network link [15].

CDs and USB interfaces can be exploited achieved through the use of an infected multimedia file. Mechanics and Engineers use the OBD port to pull the sensor and CAN bus information off the vehicle. This information is beneficial to understand the health and shape of the vehicle in great detail. It is also used to tune the vehicle and can provide firmware updates to vehicles. As such, malware can be installed using this port. Finally, an attacker can become infected by an attacker sending the malware using Bluetooth or other wireless communication links.

#### 4.3.2. Ransomware Attack

One potential malware attack on platoons is a Ransomware attack. In this type of attack, an attacker can choose to hit individual vehicles, fleet management, or the platooning service itself. In such an attack, the attacker can lock out the platooning service and even the vehicles themselves. If done on a big enough scale, such an attack has the potential to cause mass disruption. This type of attack is a genuine threat to platoons, and CAVs in general, as such attacks, are becoming more high profile with such attacks on infrastructure and hospitals making worldwide news [64,65,66].

Another way that malware can be used for financial gain is by collecting information from the vehicle. This can be done by looking directly for financial information or using information stolen from the vehicle to blackmail the user.

### 4.4. Prevent Platooning

When an attacker targets a platoon, they can do so with the intent to stop platooning from taking place altogether or at specific times. Such attacks can be targeted at specific vehicles or groups of vehicles. In such attacks, the Availability of the platooning system is compromised by the attacker as nodes cannot join or form platoons.

#### 4.4.1. Denial-of-Service (DoS) Attacks

DoS attacks can affect a platoon in one of two ways; the first is that the platoon service provider can be attacked, making it so vehicles cannot connect to them. The second is to target specific platoons. When targeting the platoon service provider, the attacker can prevent most if not all formed platoons from accepting new members, and no new platoons can be formed. This can be done by swamping the provider with more join requests than it can handle. The downside of this method is that it requires a large amount of equipment and reasonable technical knowledge to carry out.

The second method of targeting individual platoons and vehicles is very realistic. Platoons will likely have a maximum number of members that can join. This reduces the complexity of the attack as the attacker only needs to fabricate up to that many vehicles to prevent new members from joining [49]. This is because the leader will think that there are more vehicles in the platoon than there are [49]. Such attacks can be made using copied or fake vehicle IDs to connect multiple ghost vehicles to the platoon.

#### 4.4.2. Flooding Attack

Flooding attacks on platoons are where an attacker exhausts the network resources, thus preventing communications from taking place [53]. There are two types of flooding attacks: data flooding and routing control packet flooding. In data flooding, the attacker will create and transmit too many packets for the network to handle [19]. For routing control packet flooding, the attacker will send routing requests to all nearby connected vehicles regardless of whether they are part of the platoon [53]. The result is that platoon members cannot communicate with each other, thus breaking up the platoon. By performing such an attack, the attacker compromises the data verification and the availability of the network.

#### 4.4.3. Jamming Attacks

Jamming attacks can be both complex and straightforward; however, the attacker acting as a middle-man ultimately prevents a platoon from maintaining communication [9]. As platoon members cannot communicate with each other reliably, this can lead to the platoon breaking up or taking other measures to prevent an accident [9]. Jamming attacks target the Physical Layer by flooding the channels with random noise preventing platooning communications [9]. The attacker can act smartly and target individual messages or block specific channels and jamming until the platoon breaks up and then stops until the platoon reforms. When the platoon is jammed, there is a chance that a collision can occur between members. In addition, the platoon will lose any benefits it had for platooning each time it breaks up or adjusts for safety.

#### 4.4.4. Worm Hole Attack

Wormhole attacks are where two vehicles form a private communications link together and pass messages between each other. The two vehicles in question are not next to each other, and so by doing this, miss out on many vehicles [47]. Such attacks could be very problematic for very large platoons. This will cut out the vehicles between the two attackers, and manifest as a DoS attack [47]. Having two non-neighbours exchange communications as if they were neighbours will lead to the exclusion of the cut-out vehicles leading to them becoming ejected from the platoon or causing a collision. A Wormhole attack will damage the availability of the platoon.

### 4.5. Platooning Disruption

Platooning disruption attacks target platoons to disrupt and make them inefficient. Platoon disruption attacks can lead to a wide range of outcomes with the goal to prevent platoon members from gaining the benefits of platooning and or making the experience unpleasant for passengers.

#### 4.5.1. Black Hole Attacks

A Black Hole attack is one where a malicious node will receive packets from the network. The node will then not re-transmit the information to others when in a routing network [60]. By doing so, the malicious node prevents other members from receiving information in a timely manner [48,67]. As the members communicate closely together, vehicular platoon members are able to talk directly with other members. This type of attack could still severely affect communication topology that does not include a leader for everyone. The attacker could prevent messages from making it further down the platoon when using decentralised and bidirectional topology, which will lead to platoon destabilisation. By doing this, the attacker is affecting the availability of messages in the network.

#### 4.5.2. Fake Data Injection (FDI) Attacks

A fake data injection attack is when a malicious node creates a fake message and transmits it into the network [52,68]. To do so, the attacker needs to create a packet that is in the same format as the network it is transmitting into. This can be done by being a network member or copying a message format from a captured packet. Such attacks can disrupt platoons as members act upon fake information, which will cause the platoon’s stability to degrade. This will affect the traceability, data verification, and integrity of a platoon.

#### 4.5.3. Fake Position Attacks

Fake position attacks can disrupt the stability of a platoon as the attacker transmits fake position coordinates into the platoon network [19]. This misleading information will change the perceived order of the platoon, which can lead to vehicles getting messages late due to an increased routing route [19], resulting the damage to the integrity of the platoon network. In addition to routing changes and delays this can also lead to inaccurate information being used by members or even enabling the attacker to receive the information they would normally not be able to access.

#### 4.5.4. GPS and Sensor Spoofing

Platoons like CAVs have a multitude of sensors supplying information to the onboard computer about the road conditions, vehicles condition, and other traffic. In addition to this, there is also GPS for providing accurate positioning of the vehicle. Every single sensor on a vehicle can become compromised. For example, high-powered torches and lasers can blind cameras either partially or entirely [15]. In addition, there are natural and accidental threats to sensors, such as strong sunlight and dirt and dust on cameras. These will leave sensor blind spots where a vehicle may not obtain all of its typical sensor information. This can lead to the vehicle failing to react in time to a hazard, resulting in an incident.

GPS is vulnerable to both jamming and spoofing attacks, also known as tunnelling attacks [19]. Where the attacker copies the GPS transmission before replaying them, slowly moving the position away from the vehicle’s actual location. During this time, the strength of the fake signal must be stronger than the original one as GPSs are often set up to take the strongest signal as the true original message [55]. Jamming the GPS of a vehicle can be done in the same way as jamming other wireless communications. Such attacks can lead to vehicles being unable to platoon effectively, as platooning relies heavily on accurate location data to maintain coherence. Thus the attack can damage the data verification and integrity of the platoon.

#### 4.5.5. Illusion Attack

An Illusion attack is where the malicious node transmits false or misleading information into the network. In this, the malicious node will create fake messages about traffic conditions, driving conditions, and members [69]. An Illusion attack can also affect the MAC layer and disrupt the cooperation of MAC protocols. The attack can result in traffic jams, accidents, a decrease in the performance of a platoon, and degrading the integrity and data verification within the platoon network.

#### 4.5.6. Message Altering Attack

Alteration attacks target the information within a message when it is being relayed between members [57]. The effectiveness of this attack depends on the topology of the platoon. As with Black Hole attacks, this type of attack works best against decentralised and bidirectional topology as messages are routed through the attacker. The attacker could also delay the re-transmission or change the order of messages instead of changing the actual message content itself [57]. The effect of this is that member vehicles will get out-of-date or inaccurate messages, which will compromise the integrity of the network. This will lead to a reduction in the stability of the platoon as members will be reacting to old or altered messages.

#### 4.5.7. Replay Attacks

Replay attacks are where the attacker replays old messages back into a platoon [9,58]. This will, as discussed before, cause the platoon to become unstable as members react to the replayed message. The instability of the platoon can cause several problems such as significant gaps or oscillation of the platoon resulting in decreased efficiency in the platoon. Replay attacks will affect the privacy and integrity of the platoon network.

## 5. Risk Assessment in Platooning

Vehicular platoons rely heavily on wireless communication to exchange messages with each other. As shown in Section 4, there is a wide range of known attacks that can compromise wireless communications in platoons. If these attacks are successful, they can have a severe impact on the platoons since; vehicles are travelling at high speed and travelling in close formation to each other [2]. A risk assessment is used to identify the attacks that can severely impact the network, in a numeric way enabling the attacks to be ranked of risk [70]. Risk assessment is of great importance to vehicular platoons since:

1. It can list the attacks which have higher consequences on the platoons.
2. Which attacks are likely to occur within the platoons.
3. Security solutions can be designed once the attacks are ranked according to their severity.

### 5.1. Risk Analysis

SAE/ISO 21434 [12] provide guidelines for ensuring vehicle cybersecurity and is intended for use when considering the impact and risk of attacks on vehicles. Using the standard is vital in understanding the cybersecurity risk of platooning vehicles. However, the scope is limited to only the boundaries of each vehicle, making attacks such as eavesdropping out of scope as the attack takes place at the network level. Therefore, the scope needs to be expanded to include the platoon network, which is covered by ETSI TS 102 165-1 [71]. When vehicles operate as a platoon, all vehicles can be modelled as a single road user due to the compact formation, and all vehicles strictly follow the commands of the lead vehicle. Therefore in this paper, the scope of SAE/ISO 21434 is going to be extended to cover platooning behaviour and communications.

In this paper, we follow the ISO/SAE 21434 standard to conduct the risk assessment. As such, the risk of an attack in a platoon will focus on two key criteria, (a) *likelihood* (or attack feasibility) and (b) *impact*. In SAE/ISO 21434 attack feasibility means “an attribute of an attack path describing the ease of successfully carrying out the corresponding set of actions”.

Attack feasibility will take one of the four values very low, low, medium, or high, where a value of “very low” attack feasibility means that “the attack path can be accomplished utilizing very high effort”’. A value of “high” means that “the attack path can be accomplished utilizing low effort”. It is important to note that the standard does not consider the use of any countermeasures at this stage.

**Figure 13:**
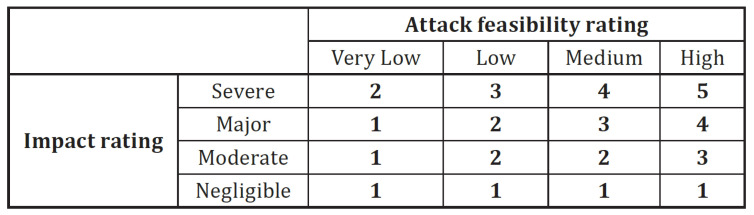
A risk matrix example.

The impact has four values negligible, moderate, major, or severe. Each of these impact values is given ratings with respect to each of the categories of safety, financial, operational, and privacy (S, F, O, P). For example, the moderate operational impact is given as “The operational damage leads to partial degradation of the vehicle function” with an example being “User satisfaction is negatively affected”. The impact value is assessed against each of these (S, F, O, P), and the most severe impact value is chosen. The risk is represented using a numerical value assigned to it by the combination of attack feasibility and impact using Figure 13 (copied from Table H.8 in ISO 21434.) It is worth noting here that the layout of the matrix is at the discretion of the OEM. It is not mandated by the standard.

### 5.2. Risk Calculations for Platoon

As SAE/ISO 21434 deals with individual vehicles, we extend our risk analysis to platoons and extend our definitions for values for platoons. For attack feasibility, we have the following definitions:

1. *Very low*: The chances of an attack are very low because the attacker will have to dedicate themselves to the attack, need to be very familiar with the vehicular platooning technology and network to carry out a successful attack as well as needing restricted specialist equipment.
2. *Low*: Here, the attacker will still need to apply themselves; however, overall, the attacker can attack without being an expert in vehicular platooning, only needing specialist equipment and a reasonable amount of time.
3. *Moderate*: Here, the attacker can be successful with a moderate understanding of vehicular platooning and related technologies, using easy-to-obtain equipment if any is needed and able to do this with little time.
4. *High*: The attacker can be successful with minimal or no understanding of platooning technologies in a very short space of time.

The *impact* of an attack is dependent on multiple factors and is the overall outcome of an attack [72]. Factors that affect the impact of an attack are safety, financial, operational, and privacy impacts of the attack [12,71]. Operational impact for platoons will also include fuel consumption, communication reliability, and road footprint [12]. The impact of an attack is the motivation to understand the corresponding risk and mitigate it to prevent the impacts from happening. We, therefore, consider the values of impact with respect to platoons:

1. *Negligible:* No physical damage to the platoon system or vehicles. No loss of sensitive data or noticeable financial loss for platoon members or providers.
2. *Moderate:* Damage to the platoon stability resulting in reduced efficiency or disbandment but no vehicle damage. Loss of sensitive data. Resulting in minor financial loss for platoon members or providers.
3. *Major:* Loss or impairment of an important platoon function, possibly resulting in a vehicle collision with a platoon member, other road user or infrastructure. Loss of sensitive data. The substantial financial loss that the platoon owner can overcome.
4. *Severe*: Loss or impairment of a core platoon or vehicle function, possibly resulting in a vehicle collision with a platoon member, other road user or infrastructure. Major loss of sensitive data leads to significant or irreversible loss. The catastrophic financial loss that the platoon owner cannot overcome.

We should note here that only one of the four categories (S, F, O, P) needs to be considered severe for the impact to be measured as severe.

### 5.3. Risks

Now that the risks have been identified, it is clear that several attacks present a critical risk to platoons as shown in Table 3. These are Flooding, Jamming, and Replay, all of which came out with a risk value of 4. As the most high-risk attacks on platoons, there must be in place countermeasures to these attacks. To understand how to prevent these attacks, it is also essential to identify the security attribute or attributes any attack compromises.

This is best done using the STRIDE threat model as it takes the threat and associates it with one or more security attributes [73]. The STRIDE threat model works well in this case as the security attributes are already defined in Section 3. The threat model can then be formed into Table 3 which shows the security attributes that are compromised by the attack.

It is important to note that there are other methods of threat models; The Process for Attack Simulation and Threat Analysis (PASTA) [74], Attack trees [75,76], LINDDUN [77], Common Vulnerability Scoring System (CVSS) [78] and many more [79].

In the below section, we provide details of the risk calculated for two attacks in vehicle platoons.

#### 5.3.1. Risk Assessment for Replay Attacks

A Replay attack’s impact rating is considered a reasonable worst-case scenario that would see the platoon start to oscillate as the old messages will give conflicting information to the member vehicles. To perform risk assessment, we made the following two crucial assumptions: (1) Multiple messages over a significant period of time are injected by the attacker, and (2) The content of the messages replayed by the attacker is very recent, i.e., injecting such messages will have consequences on the platoon network. If a message is too old, the receiving vehicle will reject the message as the time stamp or location marker will be significantly different.

The likelihood of a replay attack is considered to be likely. The attacker does not need to know very much about the technology to carry out such an attack. In addition to this, the attacker has many opportunities to strike as a replay attack can be carried out when a vehicle is connected to the platooning network. Finally, there is a small requirement for equipment; however, nothing equipment-wise is too complex or hard to acquire. Therefore the likelihood of a replay attack is high.

A replay attack’s impact is considered medium as the damage to the platoon can be extremely serious; however, it will be short-term and only while the attacker is actively attacking. Replay attacks will destabilise the platoon and lead to oscillation while some members receive repeated messages. This will reduce the efficiency of the platoon as members will not receive the full benefits of reduced air drag. There is, however, a more severe problem, and that is the chance of collisions as platoon members struggle to maintain a steady driving pattern. This will lead to an impact value of major.

#### 5.3.2. Risk Assessment for Malware Attack

Malware attacks on the platoon’s impact are again considered a reasonable worst-case scenario, just like the replay attack. In this scenario, the malware attack has managed to infect a large chunk of an operator’s fleet, locking them out of the vehicles. Equally, this could also apply to a platooning enabling company where they could have their systems taken over by the attacker.

The likelihood of such an attack is low as the attacker would need considerable time, effort, knowledge of the systems involved, equipment, and motivation to carry out such an attack. Likely, such attacks will only be carried out by major serious organised crime groups, or state actors [80]. Thus giving a replay attack a likeliness of very low.

The damage that such an attack can cause would be felt in the short term, and in the long term as such an attack can lead to a significant reputation loss. In addition to this, the company will be losing a substantial amount of money the longer the vehicles are unusable for leading to pressure to resolve the problem quickly. Malware attacks can also steal or leak sensitive information from the target during the attack, leading to an even more significant impact. This is why the impact of a malware attack is considered to be severe.

## 6. Security Mechanisms

This section aims to present and explain the range and use of security mechanisms and countermeasures proposed in the literature to the attacks identified in Section 4 in relation to platoons. In addition to presenting the countermeasures, the section will also present some of the open challenges to the use of each security method. Table 4 introduces each countermeasure and provides a small summary of what it counters and how it works, as well as a summary of the open challenge it faces.

### 6.1. Private and Public Keys

Members can use encryption keys to prevent non-member nodes from understanding messages between members. Encryption keys are broken down into two types of keys, i.e., (1) *Public key:* known by many nodes in a network or all of them. (2) *Private keys* known only by a small number of nodes that regularly communicate. This forms the public key infrastructure (PKI) [81]. For PKI to work, member nodes must agree on a common or group of common keys to use [81].

Both public and private keys work by encoding a message with predetermined algorithms, which are the keys. The keys may also add additional information to the message, such as security certificates and credentials and time stamps [6,17,47,50,81,82,83]. The additional information can be used to prevent replay attacks and give the receiver assurances on the message’s validity [17,82].

Public keys help to prevent a range of attacks on platoons such as; Eavesdrop, False Data Injection Information Theft, and False Data Message Altering [17,18,47,81,82,84]. However, public keys will only work for as long as the attacker is outside of the network. As soon as the attacker knows the public key, they can attack without hindrance.

Private keys are only shared between a small number of nodes or are even used for communications between two nodes. By doing so, private keys can prevent DoS, Sybil, and fake manoeuvre [50,82,83,84] attacks on platoons. All because the message cannot be obtained as easily as a private key is often agreed between nodes.

The challenge with keys but in particular private keys is how every node should know the key and prevent an attacker from obtaining it. One method proposed is the use of the Received Signal Strength (RSS) has been put forward as a method to use inherently random spatial and temporal variations of the reciprocal wireless channel to extract a secret key from that [50,82,83] to quickly and securely distribute private keys amongst members even in the presence of an attacker. The method works as multipath fading can be quantized, and this new digital signal can be used as a key [50,82,83]. In this way, the key is never transmitted. Furthermore, the attacker cannot obtain the key by eavesdropping. The fading is different for each receiver in the network.

Another proposed method is Convoy Protocol [11]. Here, two nodes that want to share a private key will use accelerometer data and a fingerprint extraction function to create the private key [11]. However, the method does still rely on transmitting the key to check and form an agreement on the key. The fingerprint is applied to add an element of randomness to the key and prevent an attacker from guessing the key [11].

There are other cases where sensor information is used to create private keys between vehicles, as seen in [85]. In this, a gyroscope and accelerometer are used to extract a shared private key using a fingerprint extraction function [85]. This use of two sensors makes it more challenging to replicate by the attacker than if only one sensor is used.

Another method is to use RSUs as ‘middle-men’ to distribute keys to members that request to communicate more securely together [10,84]. For this approach to work, the nodes wanting to connect must be within range of the same RSU and for both to agree to communicate. The issue with this method is that if there is no RSU in range, the private key cannot be updated or issued to the members.

### 6.2. Roadside Units

Another way to coordinate platoons and private and public keys are to use the adjacent infrastructure of the network, i.e., RSU as they can provide a contact point between platooning vehicles, road users, and companies providing platoon services [84,86]. The advantage of using RSUs is two-fold. They can serve as middle-man to communicate up-to-date information to vehicles and the Trusted Authority (TA), enabling improved connectivity. Moreover, they can monitor the driver’s behaviour within the platoon network, which can ultimately enable to detection of various attacks, including Sybil attacks [87].

They can be used to issue secret keys to individuals seeking to communicate directly with each other, such as being part of a platoon. In this capacity, the RSUs are used as intermediaries between connected vehicles and a trusted authority [19]. The RSU has limited authority. Its primary role is to distribute secret keys to authorised users to communicate with each other [84]. In some cases, the RSU is in charge of creating the secret keys. This setup gives the trusted authority much better control over who has the security key and updates the keys so that anomalous users can be screened out faster.

RSUs are still susceptible to damage, failure, and attack. The open challenge with them is identifying and removing faulty RSUs quickly and reliably, without damaging the network overall. Another open challenge is how to handle areas of the network with a low density of RSUs where platoons can not rely on them to update them from a TA.

### 6.3. Control Algorithm’s

In vehicle platooning, it is important to be able to detect abnormal behaviour of the platoon. By detecting abnormal behaviour, the vehicle can alert the driver or take corrective steps itself. The software enabling the vehicle to detect abnormal behaviour is often called control algorithms. These algorithms can reduce the impact of Sybil, replay and manoeuvre attacks as the algorithms by detecting damaging behaviours and communications caused by these attacks [54,88]. To do this, control algorithms check both sensor and communication information before the vehicle acts on it.

Platoon control algorithms can work together collectively where each vehicle is exchanging sensor information and positional information between members [88]. This information can then be filtered and statistically processed to identify and prevent potentially damaging behaviours [88].

Packet Delivery Ratio (PDR) can be used to indicate whether a platoon is being attacked [89,90]. PDR can be used to detect jamming attacks as there will be a rapid change to the PDR in the MAC layer in any given period [90]. A vehicle can be considered jammed when; the PDR rate is more than or equal to the decrease rate threshold. If the PDR value is equal to or below the PDR threshold, and finally if the PDRs decrease is positive along as the value is not equal to zero. If anyone of these conditions is met, then the node can send out a warning to others that it is being jammed [19].

Data mining has also been proposed as a way to use software to detect faulty and malicious nodes [91]. The method is called VANET Association Rules Mining (VARM). VARM works by having each node collect and temporarily store neighbouring nodes’ messages to it. The node can then extract the temporal correlation rules between itself and the neighbouring vehicles [91]. This allows the system to tell if a vehicle is defective and or malicious [91].

An Adaptive Sliding Mode Observer method has been proposed as a means to counter attacks involving the data communicated between members [92]. It is assumed that vehicles can sense the preceding vehicle’s position and velocity using frontal sensors and the intended acceleration of the vehicle using V2V communications. The approach works by using past behaviours to predict what the vehicle is going to do. That is then used to identify attacks and reduce the magnitude of the impact of the attack [92].

It is proposed that an Artificial Neural Network (ANN) can be trained to detect different types of attacks in a VANET network [93]. ANN works by analysing past communications and sensor information to make predictions about future movements. This method is just an attack detection method, which can be co-related to vehicle platoons as well.

Support Vector Machines (SVM) are shown to be useful at detecting the presence of an unknown user in a network [94]. SVM is used to share a trust value for every node in the network. The idea is that as every node in the network is aware of what is going on around it, any attackers are spotted and can be removed from the network [94].

### 6.4. Hybrid Communication

Visible Light Communications (VLC) and other wireless communication protocols can be used alongside the WAVE and the IEEE 802.11p protocol. This has been proposed as another way to improve platoon security for many attacks. VLC are well suited to their use in platooning due to the minimal inter-vehicle distance, which will reduce the likelihood that VLC can become jammed or blocked. These form hybrid communication protocols between members of the platoon [9,19,95]. Here, VLC are used as a secondary communications channel that can be used both to check and validate messages [9,19].

When VLC is used to secure a platoon, it becomes Secure Platoon Visible Light Communication (SP-VLC) [9]. In this state, both WAVE and VLC are used and transmit the same message simultaneously. When the messages are received, they are compared to ensure reliability in the message before the information is acted upon by the receiver. If one message fails to arrive, then the system can still act on just the one message [9]. This makes hybrid systems challenging to jam as it is not just one range of frequencies but two very different ones. If one system does become jammed for any reason, then the other can compensate, so the network is not jammed [9].

As the messages are checked, if there are discrepancies in the data, the system knows there is a problem [9]. At this point, the message can be rejected, or additional steps are taken to determine the real message preventing FDI and related attacks. In addition to VLC, it has also been proposed that 3GPP C-V2X communication can also be used in the same way as an alternative for non-line of sight applications [45].

### 6.5. Trust Based Security Management

Trust is an essential part of communications [96,97], and this becomes even more so when used in platooning as platooning vehicles need to work together cooperatively. Trust in platoons is a numeric value representing the reliability of the past behaviour of a platooning-enabled vehicle. In many trust-based systems, vehicles will feedback on their experience communicating with other vehicles.

Platooning trust-based systems are more dependent on having high trust values between nodes. In fact, a high trust value overall will be a highly desirable trait to a platooning node. In vehicle platooning, the trust value is almost always issued by TA [98]. The use of the TA to calculate and issue the trust values requires RSUs to collect feedback information from platooning vehicles about the vehicles they were platooning with. In VANET, however, vehicles can build up their own trust values for vehicles in close proximity to them and manage them [99,100]. This would be impractical for platoons as the extended set-up time establishing trust between members will reduce the efficiency and safety of the platoon.

Few trust models are proposed to achieve security within vehicle platoons. For instance, the REPLACE trust model presented by Hu et al. [54] relies heavily on a TA which handles requests and access to the server. The server is used to store and calculate the trust scores stored in feedback data tables. The RSUs act as an intermediary between platooning vehicles and the trusted authority. In this role, they constantly update the servers with up-to-date trust values for the trust tables. Finally, the vehicles themselves are broken down into three categories; Platoon Header vehicles, which are platoon leaders or potential platoon leaders. Potential users witch are vehicles that can become members of the platoon but are not considered platoon leaders. Finally, there are User vehicles, which are platoon members.

The goal of the REPLACE method is to create a reliable platoon recommendation service, to prevent malicious user use and abuse, and accurate judgment and evaluation of platoon leaders. To calculate trustworthiness, a Dirichlet-Based model is used to account for historical data about the trustworthiness of the vehicle enabling a quick recovery from a small one-off change in feedback, but a far much longer recovery from continuous low feedback scores. Overall the REPLACE method works well to create a database of trust values for all users. Low-trusted users could have their positions within a platoon restricted or even find themselves unable to connect. On the other side, members with high trust values are grouped, enabling members to trust each other from the start.

The Trust-based and Privacy-Preserving Platoon Recommendation (TPPR) scheme propose a way to use a trust-based system while preserving the privacy of vehicles in the network [98]. The format of TPPR is very much the same as that of REPLACE. A TA is in charge of maintaining the trust values and predicting future values based on historical data. The service provider enables the connection of the RSU together into a more extensive network and enables user feedback and trust values. In TPPR, a truth discovery-based evaluation algorithm is used to calculate the reputation scores of header vehicles. RSUs again act as an intermediary and are used to identify users in the network and relay information between the nodes and the service provider. This time, there are only two vehicles: header vehicles or platoon leaders, and the second is user vehicles which are the member vehicles.

The main difference between TPPR and REPLACE is that TPPR uses pseudonyms and the Paillier cryptosystem to improve the privacy of member vehicles. In addition to this, TPPR uses its method for evaluating the trust score of leader vehicles. The main focus of this method, however, is to preserve the privacy of member vehicles.

Vehicle platoons that use trust-based algorithms for regulating and selecting vehicles to platoon together are shown to be resistant to attacks where false or misleading information is injected into the data stream, such as FDI attacks [54,98]. Trust systems provide additional authenticity and integrity, with the trusted authority telling members whom to trust.

### 6.6. Blockchain-Based Cybersecurity

Blockchain-based methods for cybersecurity in vehicular networks is a developing area of vehicle cybersecurity that takes a decentralised and distributed approach. When applied to cybersecurity, blockchain operates as a decentralised distributed database or ledger, where the blocks are linked together to form chains [101]. It is the chains that record the information about transactions in the network. A block contains the hash value of the preceding block, one or more valid transactions, time stamps, and a random number called a nonce. Every node in the network can access a block but are unable to control the block and the information within it. Once all members have validated the block through a consensus, then it is added to the main chain [101].

Blockchain technology has several characteristics that are advantageous to vehicle platooning, are its decentralisation, consistency, audibility, good fault tolerance, and non-repudiation [101]. However, blockchain technologies do face limitations both physically and from a cybersecurity standpoint. First, blockchains face privacy issues as all data used in the auditing phase are stored publicly. Scalability can also be a problem due to the vast amounts of information that will need to be stored, particularly when operating in large networks. Another problem facing blockchain technology is that it requires mining nodes that have vast computational abilities. Finally, due to vehicles being highly mobile, this increases the computational overheads due to regular handovers [101].

## 7. Open Challenges to Vehicular Platoon Security

Platoons face many open challenges to their cybersecurity going forward, and they are presented in this section.

### 7.1. Ensuring Privacy in Vehicular Platoons

Privacy is a key consideration in cybersecurity, with consumers becoming more and more aware of their digital footprint. Platoons should be no different, with each node in the network being anonymous. However, this can create a problem as platoon topology dictates that network members need to identify themselves to know whether they need to act upon a message they receive.

While methods of preserving privacy within platoons currently do exist. The challenge is to ensure that privacy is maintained and established in the first place. The use of encryption prevents an attacker from eavesdropping once communications are established and, as such, is vulnerable to an attacker during the establishment of shared encryption. Although there are some promising methods in development as discussed in Section 4, they have not yet seen practical deployment.

### 7.2. Suitable Risk Assessment Framework in Vehicular Platoons

When considering the cybersecurity of any system, it is essential that the full risks of an attack or failure can be identified and explored. Currently, when assessing the risk for vehicle platoons, there is no single standard that can be used when considering connected vehicles, such as platooning vehicles. ISO/SAE 21434 [12] is used for cybersecurity for cyber-physical systems on vehicles; however, it does not extend to vehicle communications. Instead, ETSI TS 102 165-1 [71] standard is used to assess the risks for V2V communications. In this paper, we have met that challenge by defining platoon-based extensions to the possible values for attack feasibility and impact.

### 7.3. Lack of Suitable Real World Testbeds for Vehicular Platoons

Although platooning is starting to see limited and controlled deployment on public roads, attacks and defences are validated and tested using digital twins and not on-vehicle testing. Digital twins that are used are Plexe [102] and VENTOS [25] simulation platforms. Using such methods is very useful and can test underlying methods and theories; however, practical testing in a controlled environment can deliver more accurate real-world data. In addition to this, new attack and defence mechanisms may come to light using such practical testing. Currently, practical, real-world testbed use is still costly and requires specialist infrastructure.

### 7.4. Platoon Communication Topology Standardization

Platoon communication topology is varied, with six identified in this paper. There is the potential for many others not explicitly described in Section 2. This creates a problem when researching attacks and trying to understand the risks of attacks as the communication topology can change the attack’s impact. When investigating attack defence methods, the communication topology of the platoon is not stated. As such, it can be difficult to compare methods with each other.

### 7.5. Ransomware Attacks on Platoons

Platoons can become a vital supply chain technology to the extent that non-platooning operators may not be competitive. It is lightly that there will be sizeable platooning service operators that will provide platooning capabilities to many different companies, from independent to international.

Over the recent years, there has been a string of high-profile ransomware attackers [64,65,66]. The attackers lock a company out of their computers, preventing them from operating and stealing information. Such an attack has the potential to target a platoon service provider which could leave the fleets that they service unable to form platoons. This in itself will create massive problems for the company, including a loss of reputation [103]. The potential for the attack is to be taken one step further by locking the vehicles that have used the platooning service, preventing them from being used. The outcome of such an attack on a large enough company could affect not only local, or even national supply chains but also international suppliers [104].

### 7.6. Impact of Non-Platooning CAV’s on Platoons

Platoon technology relies on a steady, secure, and constant connection to member vehicles. As CAVs become more common on our roads and the competition for bandwidth with platoons when using the IEEE 802.11p protocol could result in a significant number of dropped and delayed packets. This could introduce additional factors that need to be taken into consideration when testing platoon security. While it is unclear in the research if test environments are populated with any other CAVs and their impact on the platoon.

In Table 3 it can be seen that attacks involving the loss, delay, or alteration of packets can be damaging to platoons. Suppose the environment already has a high packet drop rate. In that case, the platoon may be unable to use proposed countermeasures to protect against attackers.

### 7.7. Driver Behaviour (Human Factors)

Vehicular platoons, like other CAV, will need to take into consideration the human factors involved. In vehicular platoons, the human factors are similar but at the same time different from other CAVs. A vehicular platoon is controlled by a human operator overall, even if the individuals are not. This means that the platoon will be affected by the individual driving styles of the leader vehicle driver. The way that vehicular platoons handle the variations of driving styles under attack may impact the effectiveness of attacks and the proposed defences. In addition to this, it should also be investigated how driving so close together affects member vehicles’ drivers as it is a significantly smaller inter- vehicle spacing.

## 8. Conclusions

Vehicle platooning is still an emerging technology and, as such, has many unknowns. Platoon advantages have been well researched and as such, are well understood. However, as highlighted in this paper, there are still questions surrounding the cybersecurity of wireless communications in platoons. To that end, this paper has presented a current understanding of attacks to the communications of platooning vehicles and existing defence mechanisms proposed in the literature and a risk assessment of the attacks. By presenting the range of defences proposed in the literature, it is clear that there is still much debate and research into mechanisms for distributing platoon encryption keys within the network in the presence of an attacker. In addition to this, there are many proposed ways to detect potential attacks on a platoon, whether through data mining, prediction of future messages, and packet delivery ratio.

The risk assessment shows that platoons are at high risk of several different attacks which cannot be covered by a single proposed defence method. This will lead, as is recommended by the SAE standards to a defence-in-depth approach to platoon cybersecurity of wireless communications. This will lead to future research into the optimum way to apply and connect individual defence mechanisms to prevent cyber attacks on platoons through wireless communications.

Overall platooning technology will continue to develop and improve, which will require continuous research into the cybersecurity of platooning technology as new attack vectors are introduced to the technology. These attacks must be addressed before the large-scale deployment of the platoons on public roads.

## Figures and Tables

**Table 1:** Related surveys addressing cybersecurity of Platoons, CAV, and VANETs. A detailed discussion of a topic is denoted with a ✓, No discussion uses a ✕ and limited discussion is Lim.

Paper	Attacks on Platoons	Security Attributes Broken by Attacks	Connected Vehicle Cyber Defences	Risk Assessment
Amoozadeh et al. [13] 2015	Lim	✕	Lim	✕
Jia et al. [14] 2015	Lim	✕	Lim	✕
Parkinson et al. [15] 2017	Lim	Lim	Lim	✕
P.K. Singh et al. [16] 2018	✓	✕	✕	✕
Lu et al. [17] 2019	✕	✓	✓	✕
Huang et al. [18] 2020	Lim	✓	✓	✕
Ghosal et al. [19] 2021	✓	✓	✓	✕
Balador et al. [20] 2022	Lim	Lim	Lim	✕
Current Paper 2022	✓	✓	✓	✓

**Table 2:** Threats to platoons and a summary of how the attack will compromise the platoon.

Attack Name	Short Summary of the Attack	Goal of the Attack
Black Hole [48]	Compromises the Availability by not passing on messages to other members.	Platoon Disruption
Collision attacks [19]	Compromises the Availability as the attacker deliberately courses message collisions and controls what packets are transmitted.	Access Management
Denial Of Service [49]	Compromises the Availability of the network by preventing users from joining or creating a platoon.	Prevent Platooning
Eavesdropping [50]	Compromises the Confidentiality of the network because an attacker can understand the information transmitted within the platoon. This can lead to data theft and privacy violation.	Data Collection
Fake Maneuver attack [39,51]	Compromises the Integrity of the network by creating fake manoeuvre requests for members in the platoon. This will destabilise and prevent users from using the platoon by breaking it into smaller platoons or creating entrance gaps for nonexistent vehicles. Members can also be removed.	Platoon Disruption
False Data Injection [52]	Compromises the traceability, data verification and integrity of the platoon as the attacker is able to inject fake messages to manipulate the platoon behaviour to there advantage	Platoon Disruption
Fake position attacks [19]	Compromises the integraty of the platoon as the attacker reports to be in a diffrent position in the platoon.	Platoon Disruption
Flooding [53]	Compromises the Availability and Data Verification as the attacker overwhelms the network with more messages or data than is can handle.	Prevent Platooning
Illusion [19]	Compromise the Data Verification and Integrity of the platoon network as the attacker creates one or more ghost vehicles.	Platoon Disruption
Impersonation [54]	Compromises the Integrity of the network by an attacker posing as a different individual in the network. This leads to false representation and reputation damage.	Access Management
Information Theft [15]	Compromises the platoons Privacy as the attacker is able to capture data from platoon members.	Data Collection
Jamming [9]	Compromise the Availability of the network as an attacker seeks to prevent all communications on platoon frequencies in the local area. As platoon members can no longer communicate, it will disband.	Prevent Platooning
Jamming and Spoofing Sensors [15,55]	Compromises Authenticity and Availability of sensors. This is done using malware or directly attacking the sensor, which will lead to false sensing.	Platoon Disruption
Location attacks [56]	Compromise the Confidentiality and Privacy of members in the platoon as the attacker can identify individuals locations.	Data Collection
Malware [15,54]	Compromises the Availability of the network by preventing users from being able to platoon. However, malware can also carry out other attacks such as data theft, sensor spoofing and DoS attacks on the vehicle itself.	Financial Gain
Message Altering [57]	Compromises the Integrity of messages in the platoon as the message content has been changed.	Platoon Disruption
Replay [9,58]	Compromise the Integrity of the network as an attacker replays old messages into the network. This makes the platoon unstable as members receive conflicting information.	Platoon Disruption
Repudiation [19]	Compromises the Authority and Non-repudiation of the network as the attacker will deny receiving messages.	Access Management
Sybil attack [10,54]	Compromises authentication of the network by an attacker within the platoon making ghost vehicles that will try to get accepted into the platoon. This leads to destabilisation and prevents members from joining.	Access Management
Worm Hole [47]	Compromises the Availability of the platoon by cutting out members of the platoon.	Prevent Platooning

**Table 3:** Complete table of attack risks also shows which security attribute is broken by each attack. with Au being Authenticity, Av being Availability, C being Confidentiality, D being Data Verification, I being Integrity, P being Privacy, Ne being Network User Management and No being Non-repudiation.

Attack	Attack Feasibility	Impact	Risk	Security Attribute						
				Au	Av	C	D	I	P	No
Collision	2	2	2		✓					
Impersonation	2	3	2	✓	✓					
Maneuver	2	2	2		✓			✓		
Repudiation	3	3	3	✓						✓
Sybil	1	4	2	✓			✓			
Eavesdropping	3	2	2			✓			✓	
Information Theft	3	3	3			✓			✓	
Location Tracking	4	1	1			✓			✓	
Malware attack	1	4	2		✓	✓		✓	✓	✓
Denial Of Service	3	3	3		✓			✓		
Flooding	4	3	4		✓		✓			
Jamming	4	3	4		✓					
Worm Hole	2	3	2		✓					
Black Hole	3	3	3		✓					
Fake Data Injection (FDI)	2	4	3				✓	✓		
Fake Position	2	2	2				✓	✓		
GPS and Sensor Spoofing	1	2	1				✓	✓		
Illusion	2	3	2				✓	✓		
Message Altering	3	3	3				✓	✓		
Replay	4	3	4					✓	✓	

**Table 4:** Vehicular Platoon defence methods identified from the literature.

Security Mechanism	Security Attribute Secured	Open Challenge
Secret and Public Keys	Authentication, Confidentiality, Integrity and Privacy	Large scale testing of current methods of key creation and distribution to compare effectiveness against the cost.
Roadside Units (RSU)	Availability and Data Verification	More research into RSU network deployment and identification of rouge RSUs.
Control Algorithms	Authentication, Data Verification, Integrity and Non-repudiation	Where in the network is the most efficient to deploy and use the algorithms.
Hybrid Communications	Availability, Data Verification and Integrity	The use of VLC and wireless radio communications between V2I is lacking
Trust-Based methods	Authentication, Confidentiality and Integrity	Requires connection to a trusted authority for management and distribution of trust values.
Blockchain	Authentication, Data Verification, Non-repudiation and Integrity	Reducing computational power required for large networks and maintaining privacy.

## Abbreviations

Acronyms and their description:

**Acronym**	**Description**
ACC	Adaptive Cruise Control, which is when a vehicle’s cruise control is able to make adjustments based on sensor feedback.
CAN	Controller Area Network is a network connecting different sensors and onboard Computers within the vehicle.
CAV	Connected and Autonomous Vehicle, Any vehicle that is connected to other vehicles or infrastructure and or is an autonomously driven vehicle.
CACC	Cooperative Adaptive Cruise Control is a cooperative extension of adaptive cruise control.
DoS	Denial of service preventing users to provide or access services.
FDI	Fake Data Injection, transmitting fake or tampered messages into the network.
GPS	Global Positioning System, satellite-based modern computerised navigation tool.
ID	Identification, a unique code given to the vehicle.
IoT	Internet of Things are networks created by physical objects exchanging data.
LiDAR	Light Detection and Ranging, using infrared light to create a 3D view of the world.
PKI	Public Key Infrastructure, set of rules for secure communication using public and private keys.
RSU	Roadside Unit, static entity along the roadside to provide connectivity to vehicles and Trusted Authority (TA).
SP-VLC	Secure Platoon Visible Light Communication, visible light communications used specifically to secure a platoon.
TA	Trusted Authority, entities in the infrastructure domain to provide trusted services.
VANET	Vehicular Ad hoc NETwork, transportation network where two vehicles communicate via V2X wireless communication technology.
VPD-ADA	Vehicular Platooning Disruption attack detection algorithms designed to detect malicious code or communications which disrupt the natural flow of a platoon
VPD	Vehicular Platoon Disruption, anything that disrupts the natural flow of a platoon.
V2I	Vehicle-to-Infrastructure communications between vehicles and nearby infrastructure, such as RSU.
V2V	Vehicle-to-Vehicle communications between two or more vehicles.
V2X	Vehicle-to-Everything communications, between vehicle and every network entity.
WAVE	Wireless Access for Vehicular Environments, is the way that wireless communications are carried out in vehicle communications in accordance with the IEEE 1609 Family of Standards.
